# Endoscopic endonasal approach for acromegaly: surgical outcomes using 2018 consensus criteria for remission

**DOI:** 10.20945/2359-3997000000650

**Published:** 2023-06-19

**Authors:** Rodrigo Alves de Carvalho Cavalcante, Luiz Alves Vieira, Luís Felipe Araújo Peres, Alice Jardim Zaccariotti, Helioenai de Sousa Alencar, Estela Muszkat Jatene, Leandro Azevedo Camargo, Monike Lourenço Dias Rodrigues

**Affiliations:** 1 Universidade Federal de Goiás Faculdade de Medicina Hospital das Clínicas Goiânia GO Brasil Departamento de Cirurgia Neurológica, Hospital das Clínicas, Faculdade de Medicina, Universidade Federal de Goiás, Goiânia, GO, Brasil; 2 Hospital do Câncer Araújo Jorge Goiânia GO Brasil Serviço de Cirurgia Neurológica, Hospital do Câncer Araújo Jorge, Goiânia, GO, Brasil; 3 Universidade Federal de Goiás Faculdade de Medicina Goiânia GO Brasil Faculdade de Medicina, Universidade Federal de Goiás, Goiânia, GO, Brasil; 4 Universidade Federal de Goiás Faculdade de Medicina Hospital das Clínicas Goiânia GO Brasil Departamento de Endocrinologia, Hospital das Clínicas, Faculdade de Medicina, Universidade Federal de Goiás, Goiânia, GO, Brasil; 5 Universidade Federal de Goiás Faculdade de Medicina Hospital das Clínicas Goiânia GO Brasil Departamento de Otorrinolaringologia, Hospital das Clínicas, Faculdade de Medicina, Universidade Federal de Goiás, Goiânia, GO, Brasil

**Keywords:** Acromegaly, endoscopic endonasal, transsphenoidal surgery, surgical outcomes

## Abstract

**Objective:**

The primary aim is to analyze the endoscopic endonasal surgical results in short-term and two-year follow-ups according to the 11th Acromegaly Consensus statement (2018). Indeed, prognostic factors and complications were analyzed.

**Subjects and methods:**

40 patients who underwent endoscopic endonasal surgery by acromegaly between 2013 to 2020 was analyzed. Patients were considered in remission if an upper limit of normal (ULN) IGF-1 was less than 1.0 at the six-month and two-year follow-ups. Moreover, we assessed the Knosp grade, tumor volumetry, ULN, T2 signal in MRI, reoperation, and complications.

**Results:**

The mean age of admission was 46.7 years. Thirty-two patients were in remission after six months of surgery (80%), decreasing to 76.32% at the two-year follow-up. All microadenomas presented remission (n = 6). Regarding the complications, three patients had permanent panhypopituitarism (7.5%); postoperative cerebrospinal fluid (CSF) leaks did not occur in this series. The hyperintense signal on the T2 MRI and a higher tumor volumetry were the single predictor's factors of non-emission in a multivariate regression logistic analysis (p < 0.05). Preoperative hormone levels (GH and IGF-1) were not a prognostic factor for remission. The re-operated patients who presented hypersignal already had a high predictor of clinical-operative failure.

**Conclusion:**

The endoscopic endonasal surgery promotes high short-term and two-year remission rates in acromegaly; the tumor's volumetry and the T2 hypersignal were statistically significant prognostic factors in non-remission – the complications presented at similar rates in comparison to the literature. In invasive GH-secreting tumors, we should offer these patients a multi-disciplinary approach to improve acromegalic patients’ remission rates.

## INTRODUCTION

Pituitary adenoma accounts for up to 15% of all primary intracranial tumors. Acromegaly is a chronic systemic disease caused by an excessive growth hormone (GH) and insulin-like growth factor 1 (IGF-1) secretion, in a substantial majority due to a GH-secreting pituitary adenoma (1).

Acromegaly has an estimated prevalence and annual incidence of 59 and 3.8 cases per million inhabitants per year, respectively (2). Therefore, the diagnosis is often made during life's 3rd to 4th decade (3). The key clinical features are acral enlargement, coarse face, headaches, arthropathy, hyperglycemia, cardiomyopathy, and sleep apnea. In addition, the disease increases the standardized mortality ratio (SMR) in 1.7 of these patients compared to the general population (4). The diagnosis is confirmed by elevated GH and IGF-1 levels in patients with a specific clinical picture, followed by a pituitary MRI to determine the source of the excess GH (5). Hormonal control results in the reduction of symptoms and mortality rates; hence, the acromegaly treatment aims to normalize IGF-1 levels, according to the 11th Acromegaly Consensus Conference (6).

The treatment options for acromegaly include surgery, medical treatment, and radiosurgery. Surgical resection has been considered the first-line treatment for acromegaly, mainly in pituitary tumor centers of excellence (PTCOE) (7). In this context, the endoscopic endonasal approach has been the best approach for most pituitary tumors. It promotes a broad view, facilitating the resection of tumors in the suprasellar area and the cavernous sinus. The overall biochemical remission rates in endoscopic series vary from 38% to 85% using previous consensus criteria (8). This study aims to analyze endoscopic endonasal surgical outcomes in short-term and two-year follow-ups, predictors of remission, and complications of acromegalic surgically treated patients from a single tertiary center according to the 11th Acromegaly Consensus Statement (2018) (6).

## SUBJECTS AND METHODS

All acromegalic patients who underwent surgery between January 2013 and December 2020 had their data retrospectively analyzed. In this sample, of 44 operated patients, four had incomplete data and were excluded.

The diagnosis was defined based on proportionately high growth hormone (GH) and insulin-like growth factor (IGF-1) associated with the contrast enhanced tumors detectable on pituitary resonance imaging (MRI). The IGF-1 upper limit of normal (ULN), calculated by using the patient IGF-1/upper limit of normal IGF-1 for age and sex, was considered high when ≥ 1.00. Several laboratories assessed GH and IGF-1; however, IMMULITE 2000 (Siemens Healthineers, Malvern, PA, USA) chemiluminescent methods are the predominant GH and IGF-1 laboratory kits in the state of Goiás, Brazil, where all patients reside. Moreover, IGF-1 ULN calculation makes IGF-1 assessments comparable among patients.

The contrast T1-weighted and T2-weighted MRI acquisitions were performed. An experienced neuroradiologist (especially regarding modified Knosp and T2 signal – hypointense or hyperintense relative to the adjacent pituitary gland or the grey matter of temporal cortical signal) suggested densely or sparsely granular tumors, respectively (9). About re-operated patients, the T2-MRI signal was analyzed based on the MRI performed before their first surgery. Tumor volume was calculated using the Di chiro-Nelson formula (V= 1/2 H. W. L); H – the height of hypophysis or craniocaudal size (cm); W – width or lateral size (cm); L – the length of hypophysis or anteroposterior size (cm) (10). The modified Knosp classification to estimate the cavernous sinus invasion is shown in Table 1. We divided the modified Knosp into two groups: Knosp 0, 1, 2 (non-invasive tumors) and Knosp 3A, 3B e 4 (invasive tumors).

**Table 1:** Knosp modificated (11)

Grade	Definition
Grade 0	No invasion, all the lesion medial to the cavernous carotid artery
Grade 1	Invasion extending to, but not past, the medial aspect of the cavernous carotid artery
Grade 2	Invasion extending to, but not past, the lateral aspect of the cavernous carotid artery
Grade 3A	Invasion past the lateral superiorly aspect of the cavernous carotid artery, but not completely filling the cavernous sinus
Grade 3B	Invasion past the lateral posteriorly aspect of the cavernous carotid artery, but not completely filling the cavernous sinus
Grade 4	Tumor completely filling the cavernous sinus both medial and lateral to the cavernous carotid artery

The primary outcome was defined as biochemical remission according to the 2018 Acromegaly consensus criteria; In this statement, IGF-1 normalization best reflects disease control. IGF-1 levels were evaluated after correcting eventual thyroid and steroidal former postoperative deficits at the six-month and two-year follow-ups. The senior surgeon (RACC) performed all surgical procedures after a careful endocrinological workup. Out of that, nine patients had undergone surgery in another medical facility using the sub-labial transsphenoidal microscopic approach. They were under somatostatin analogs medicine after this approach. The other 31 patients had not been treated with medication for acromegaly. Besides, acromegaly-related comorbidities (such as systemic arterial hypertension, Mellitus Diabetes, and malignant disease) were set down. Normal IGF-1 levels for age and sex and ULN < 1.0, at the six-month and two-year follow-ups, with no somatostatin analogs or cabergoline use, associated with no visible pituitary tumor MRI defined the remission state in a short-term and two-year follow-ups.

### Statistical analysis

The Kolmogorov-Smirnov test of normality defined sample distribution. Due to Gaussian distribution, parametric comparisons were made between groups of two by two using paired t-tests. The Wilcoxon test analyzed modified Knosp and the presence of remission. In the unpaired analyzes between different groups, the Mann-Whitney test was used (Wilcoxon rank-sum test). The χ^2^ test (Chi-square) and corresponding Fisher test were used for risk verification. Pearson's correlation coefficient correlated the different tests for the response pattern taking IGF-1 and the direct measure of the ULN adjusted for sex and age as the standards. The evaluation of remission predictors was analyzed by binomial logistic regression analysis. A p-value < 0.05 was considered statistically significant. All analyzes were performed by R Software, version 4.2 (The R Foundation, USA).

## RESULTS

Of 40 patients, 21 patients (52,5%) were female. The mean age at surgery was 46.7 ± 13.1 years (19-73 years). Patients were follow-up with from 6 to 63 months (41.5 ± 21.63 months).

Nine patients (22.5%) underwent a previous surgery in another facility; however, these patients presented a residual lesion at the postoperative pituitary MRI. Thus, they were placed on medical treatment using somatostatin analogs for acromegaly. The remaining 31 patients (77.5%) had not experienced any earlier medical or surgical treatment. Regarding the lesion size, 85% (n = 34) were macroadenomas and 15% (n = 6) were microadenomas. The recorded volume was 1.45 ± 13.64 cm³ (0.034-28.8 cm³). Concerning the modified Knosp score (Table 1), lesions were evaluated at T1 gadolinium-enhanced MRI with the identification of 16 patients in grade 0 (40%), nine patients in grade 1 (22.5%), nine patients in grade 2 (22.5%), three patients in grade 3A (7.5%), one patient in grade 3B (2.5%) and two patients in grade 4 (5%) (11). Hence, 34 (85%) patients harbored non-invasive tumors (Knosp 0, 1, and 2), and six patients (15%) had invasive tumors (Knosp 3A, 3B, and 4). Of the 40 patients, 14 had a hyperintense signal on the T2 MRI (35%), as summarized in Table 2.

**Table 2:** Demographic characteristics of the series studied in relation to the parameters analyzed

Patients characteristics		
Female	21 (52,50%)	
Male	19 (47,50%)	
Age	51 ± 13,69	
Previous surgery	9	
	**Possible remission predictors**	
	**Remission (n = 32)**	**Non-remission (n = 8)**
Age at surgery	47,5	43,5
Hypersignal	9 (28,12%)	5 (62,50%)
Hyposignal	23 (71,88%)	3 (37,50%)
Macroadenoma	26 (81,25%)	8 (100%)
Microadenoma	6 (18,75%)	0
		**Percentage of non-remission based on Modified Knosp**
**Non-invasive**		
0	15	1 (6,2%)
1	7	1 (12,5%)
2	7	2 (22,2%)
**Invasive**		
3A	2	1 (33,3%)
3B	1	1 (50%)
4	0	2 (100%)

Regarding hormonal assessments obtained through chemiluminescence, the preoperative GH levels were 34.29 ± 125.16 μUI/L (0.4-800 ng/mL), whereas IGF-1 levels had 599.60 ± 244 ng/mL (236-1309 ng/mL). The IGF-1 ULN had a mean of 2.52 ± 1.10 (0.98-5.76). Furthermore, 16 patients (40%) had Mellitus diabetes that impairs the oral glucose tolerance test (OGTT) – GH assessment.

After surgery, GH levels at six months dropped to 1.27 ± 1.71 ng/mL (0.05-10 ng/mL), while IGF-1 levels of 217 ± 136 ng/mL (81-742 ng/mL), (p < 0.05). The calculated ULN was 0.92 ± 0.61 (0.37-3.86). The IGF-1 mean was 203 ± 131 (0.15-545), and the calculated ULN means kept at lower levels of 0.89 ± 0.58 (0.2-3.2), p < 0.001 at two-year follow-up.

### Remission data

Eight patients (20%) did not achieve hormonal remission after six months, and 32 were in remission (80%). At two-year follow-up the remission rate decreased to 76.32%. Two patients died one-year after surgery due to COVID-19 and one patient died by non-acromegaly related diseases. One patient relapsed during this period of two-year follow-up after short-term remission. As for the established remission criteria, both GH and IGF-1 were equally effective (p = 0.80).

Two reoperations showed no remission (22.22% of reoperations). Patients who had not undergone previous surgery had a remission rate of 80.64% (25 of 31), against 77.78% of reoperations (7 patients).

Eight macroadenomas (23.52%) do not present remission. Meanwhile, all microadenomas presented remission.

### Prognostic factors

**Figure 1:**
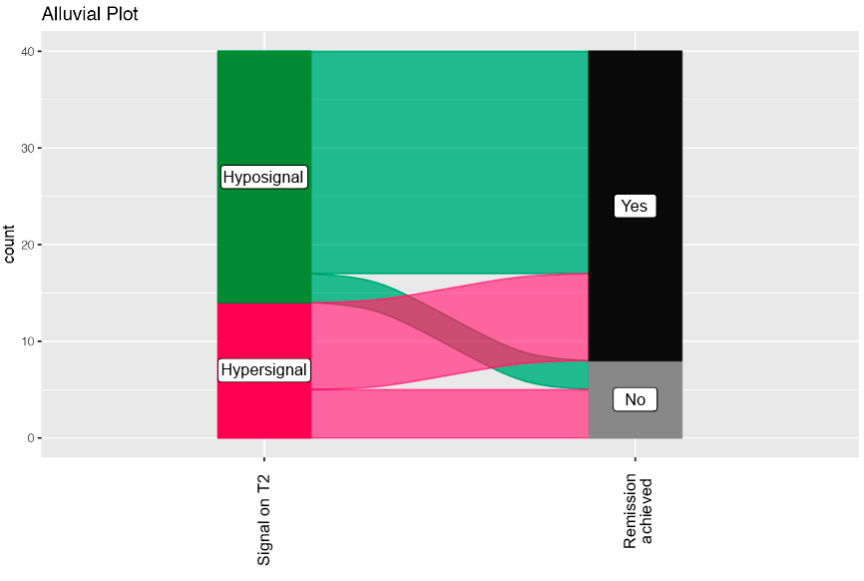
Alluvial diagram demonstrating relationship between T2 signal in MRI and remission.

Firstly, we performed a univariate logistic regression analysis model considering GH and IGF-1 preoperative levels, tumor volume, reoperation, the signal on T2-weighted MRI, gender, and modified Knosp grade. The p-value was statistically significant if p < 0.2. In the initial model, only signal on T2-weighted MRI, gender, modified Knosp grade, and volume was predictive of remission. In the final logistic regression analysis model, only a higher tumor volume and hyper signal on T2-weighted MRI were predictors for non-remission (Table 3). The hyperintense signal on the T2 MRI was related to a lower remission rate (5 patients, 37.5% in the subgroup) with p < 0.05, as shown in table 3 (p < 0.05). Of the 26 patients with a T2 hypointense signal, only three did not present remission, 11.5% within the subgroup (Figure 1).

**Table 3:** Multivariate analysis using by binomial logistic regression of remission with respect to tumor volume and T2 signal

		95% Confidence Interval		
Predictor	Odds ratio	Lower	Upper	P
Volume (cm³)	1.153	1.0191	1.306	0.024
Hyperintense signal on T2	0.102	0.0112	0.941	0.044

Measures of model fit: Adjusted R2 0.325; χ^2^: 13.0; p: 0.002.

Table 3 summarizes the variables regarding the statistical analysis in the binomial logistic regression based on remission with their respective confidence intervals.

The regression analyzes demonstrate a compelling joint effect of reoperation with hypersignal as factors strongly associated with surgical failure, which cannot be evidenced in the isolated analysis of reoperation. Re-operated patients who presented hypersignal already had a higher prediction of clinical-operative failure.

Additionally, we did not find a significant statistical association between hyposignal on T2 MRI and non-invasive tumors – Knosp (0, 1, 2) (P > 0.05); there was no association found between smaller tumors and hyposignal on T2-MRI using a Chi-square test.

### Complications

Three patients developed permanent postoperative panhypopituitarism (7.5%). Epistaxis, transient abducens paresis, cerebrospinal fluid (CSF) leak, meningitis, recurrent rhinosinusitis, and persistent diabetes insipidus occurred once in six isolated patients (2.5% each). There were no procedure-related postoperative deaths during data recording (30 days to 2 years).

**Figure 2:**
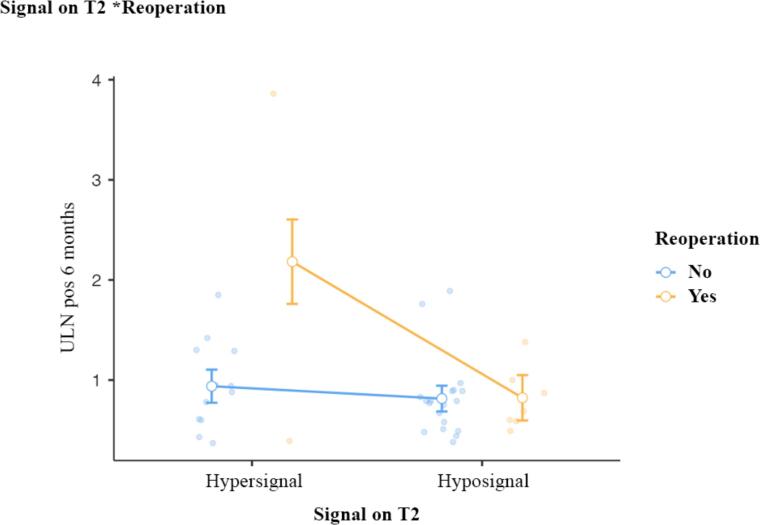
Signal covariate interaction analysis on T2 in the reoperated and non-reoperated groups based on postoperative ULN level.

Table 3 summarizes the variables regarding the statistical analysis in the binomial logistic regression based on remission with their respective confidence intervals. Figure 2 shows the analysis of covariance.

The regression analysis demonstrates a compelling joint effect of reoperation with hypersignal as factors strongly associated with surgical failure, which cannot be evidenced in the isolated analysis of reoperation, however, maintained in the isolated analysis of signal on T2 (Figure 2). Re-operated patients who presented hypersignal already had a higher prediction of clinical-operative failure.

## DISCUSSION

In recent decades, endoscopic endonasal treatment of sellar lesions has proven to be a viable option for lesions that extend beyond the midline (12). Its importance portrays better exposure, more panoramic and instrument maneuverability, ability to look around anatomical corners using angled lenses, less nasal trauma, greater patient comfort, and better results concerning more extensive tumor resection (13). Thus, the endoscopic technique may be preferred, and it is suggested that more than 90% of pituitary tumors, mainly invasive GH – secreting macroadenomas, should be treated through this approach (14,15).

We found an overall surgical remission of 76% and 100% for macroadenomas and microadenomas, respectively, using the 2018 consensus criteria for remission in acromegaly. This result agrees with other endoscopic series (8). In these series, the surgical remission rates vary from 67%-100% for microadenomas and 55%-80% for macroadenomas using different consensus criteria (2000 and 2010). We considered six-month follow-up remission patients with normal for age and sex IGF-1 and < 1.0 IGF-1 ULN. When choosing the six-month, the authors tried to avert the delayed decline of IGF-1 levels after surgery, reaching 24% of patients in the final analysis (16). As the 2018 consensus recommended, a key goal for remission is the normalization of IGF-1 levels. As suggested, we did not consider postoperative GH levels in this analysis for remission due to our practice's lack of ultrasensitive GH assays, pulsatile nature, and multiple GH assay kits. Moreover, 40% of our patients had mellitus diabetes which impairs oral glucose tolerance test (OGTT) – GH evaluation.

Analyzing predictors of remission, we did not find predictions of remission for preoperative GH and IGF-1 levels. On the other hand, the volumetry, and the hyper signal on T2 weighted MRI were predictors for non-remission using binomial logistic regression analysis.

Preoperative GH and IGF-1 levels are considered predictors for remission in some series (17,18). However, we did not find this correlation like Antunes and Yildirim (19,20). Concerning factors associated with non-remission: tumor volumetry is a controversial topic; some series show that higher volume tumors are isolated predictors for remission, in the same way as our study. In other series, they have not observed such a difference. The origin of this controversy is based on a lack of analysis of cavernous sinus tumor invasion in some studies.

As for cavernous sinus invasion, our study demonstrated that tumors with Knosp more than 3B have more propensity to non-remission, like many other studies (1,8,21). Recently, Mohyeldin and cols. raised questions about the reliability of the Knosp score in predicting an actual invasion of the medial of the cavernous sinus by pituitary adenomas, mainly for acromegalic patients. Their study found invasion in the medial wall in 25%, 67%, and 100% for Knosp 0, 1, 2, 3, and 4, respectively (22). This finding suggests that the medial wall resection should be considered for acromegalic patients once the authors reach remission rates of 92% using this maneuver. This topic is still controversial. Nonetheless, it could explain why we have not achieved remission in four patients with Knosp 1 and 2 despite gross total tumor removal in the intraoperative analysis and postoperative MRI scan.

The hypointense signal on the T2 MRI was highly related to remission: in 23 of 26 patients (88.5% within the subgroup), as seen in the Alluvial Plot (Figure 1). Besides, the hyperintense signal was related to a lower remission rate (5 patients, 37.5% in the subgroup) with p < 0.05. Our study is the first in the literature to report the hyper signal on T2-MRI as a predictor for non-remission. This finding can help us to predict remission using pre-surgical imaging. Potorac and cols. have shown that T2-hypointense tumors are less likely to invade the cavernous sinus and have densely granulated patterns. Those characteristics may implicate a better response to somatostatin ligand therapy and more significant tumor shrinkage (9,20,23). On the contrary, analyzing the T2 signal, we found just a single author that evaluated this correlation. Antunes’ article did not find a correlation between the signal on T2 and surgical remission (20).

Meanwhile, the remission rate on reoperation was 77.78% (7 of 9), and for those who had not undergone previous surgery was 80.64% (25 of 31). It was noted as statistically significant in the covariance analysis (p = 0.0032). Concurring with others studies (24-27), surgical results are generally less favorable than in primary operations. Furthermore, some complications increased in reoperations for pituitary tumors because of tissue defects that distort the anatomy and postoperative sequelae, making surgery technically more difficult.

Nevertheless, surgery complications such as epistaxis, transient abducens paresis, meningitis, recurrent rhinosinusitis, and persistent diabetes insipidus (2,5%) were not typical in our series. In addition, one patient had postoperative CSF leaks (2,5%), and three patients had permanent panhypopituitarism (7.5%). The reported incidence of rhinorrhea in the postoperative period of endoscopic pituitary surgery generally varies between 0.7% and 12%, and the risk of permanent diabetes insipidus with endoscopic surgery was 0.7% to 8.5%. Moreover, carotid artery injury is a rare but severe and potentially fatal complication reported at less than 1% (28).

On the other hand, Leopoldo and cols. series (23 patients evaluated for GH-secreting adenomas resection) found complications in 56,5% of patients: 8,7% with permanent diabetes insipidus, 17.4% with otorhinolaryngologic complications, 4,3% with meningitis, 21.7% with CSF leaks, and 8.7% developed new panhypopituitarism (14). Gondim and cols. (67 cases) found epistaxis (6.0%), transitory diabetes insipidus (4.5%), one case of seizure (1.5%) and five cases of permanent thyroid hormone deficiency (7,5%) (13).

As noted, our series had a low complication rate. We can assume that this reflects our objective diagnostic criteria and preoperative imaging, besides optimal and adequate surgical technique. As was also seen in the series by Hazer and cols. in which it was essential for the first surgery to be performed by an experienced senior surgeon, thus contributing to higher cure rates (12).

Although the authors have presented one of the most extensive series in our country of endoscopic endonasal surgery for acromegaly, our study has some limitations due to the sample size and retrospective design.

In conclusion, the endoscopic endonasal surgery promotes high short-term and at two-year remission rates in acromegaly. The tumor's volumetry, and the T2 hypersignal, were statistically significant prognostic factors in non-remission. Complications presented at similar rates in comparison to the literature. In invasive GH secreting tumors, we should offer these patients a multi-disciplinary approach to improve acromegalic patients’ remission rates.
